# Personality associations with Facebook use and tendencies towards Facebook Use Disorder

**DOI:** 10.1016/j.abrep.2020.100264

**Published:** 2020-02-19

**Authors:** Cornelia Sindermann, Éilish Duke, Christian Montag

**Affiliations:** a Department of Molecular Psychology, Institute of Psychology and Education, Ulm University, 89081 Ulm, Germany; b Department of Psychology, University of Huddersfield, Queensgate, Huddersfield HD1 3DH, UK

**Keywords:** Facebook, Big Five, Personality, Facebook Use Disorder, Internet Communication Disorder, Social Networks Use Disorder

## Abstract

**Introduction:**

The present study sought to address core issues in the association between personality and the putative disordered use of Facebook. First, to redress the issue of generalisation from samples solely recruited from Facebook, we sought to explore personality differences between users and non-users of Facebook. Second, we aimed to investigate associations between personality and Facebook Use Disorder. The present study contributes a novel perspective to extant research on this topic by moving beyond the broad Big Five of personality, to explore possible relationships between Facebook use and sub-facets of the Big Five; all analyses were additionally controlled for confounding effects of demographic variables.

**Methods:**

3,835 (*n* = 2,366 males) participants completed socio-demographic variables, the Big Five Inventory and stated their user status on Facebook (i.e. user versus non-user). Facebook-users also completed a Facebook Use Disorder scale assessing addictive tendencies towards Facebook use.

**Results:**

Facebook users reported higher levels of extraversion and lower levels of conscientiousness compared to non-users. Tendencies towards Facebook Use Disorder correlated negatively with conscientiousness and positively with neuroticism in both males and females.

**Conclusions:**

The present results indicate that research samples drawn from Facebook users may be biased with regard to personality (extraversion, conscientiousness). Moreover, certain personality traits – conscientiousness and neuroticism – which may influence the tendency towards Facebook Use Disorder are discussed.

## Highlights

• Facebook users reported higher levels of extraversion compared to non-users.
• Facebook users reported lower levels of conscientiousness compared to non-users.
• Tendencies towards Facebook-Use Disorder correlated negatively with conscientiousness.
• Tendencies towards Facebook-Use Disorder correlated positively with neuroticism.
• All results are controlled for potential effects of demographic variables.

## 1 Introduction

### 1.1 General introduction

Use of online social networks (SNs) such as Facebook, Twitter, and Instagram is an increasingly common aspect of modern life. Facebook, which boasts approximately 2,375 million active users, is one of the largest SNs worldwide (We Are Social, Hootsuite, & DataReportal, 2019). Reflecting this popularity, the Facebook application is one of the most frequently downloaded social (networking) apps from Apple’s app store and Google’s Play store, suggesting that a large proportion of people have constant access to Facebook via their smartphone (Priori Data, 2019a, Priori Data, 2019b).

Given its popularity, Facebook affords researchers access to a large number of prospective participants. However, sole reliance on Facebook as a recruitment platform may lead to biases in sampling, particularly if robust individual differences, e.g. in personality traits, exist between users and non-users. Personality can be defined as “stable individual differences in cognitive, emotional and motivational aspects of mental states that result in stable behavioral action (especially emotional) tendencies of humans …” (Montag & Panksepp, 2017, p. 1). Given this definition, it seems logical that certain personality traits may be associated with whether or not people use Facebook. Therefore, if only recruiting Facebook users, such differences between users and non-users may lead to the publication of data that cannot be generalised beyond the sample of Facebook users (Brickman Bhutta, 2012, Thornton et al., 2016, Whitaker et al., 2017). It is, therefore, important to investigate potential systematic differences between Facebook users and non-users in personality, which was the first aim of the present study.

Next, potential addictive tendencies towards the use of Internet activities such as the use of SNs like Facebook have been investigated in several studies (see for example studies mentioned in paragraph 2.2). Specifically, the putative addictive tendencies toward the Internet have been parsed into generalised and specific activities (Davis, 2001). According to Davis’ model, specific Internet Use Disorders do not refer to the disordered use of the Internet in general, but to the disordered use of specific activities, such as gambling, gaming, shopping, pornography, and social networking/communication on the Internet (Brand et al., 2016, Davis, 2001, Montag et al., 2015, Müller et al., 2017). Addictive tendencies towards SNs as a specific form of Internet Use Disorder are often referred to as “Social Network Site Addiction”, “Internet Communication Disorder”, or “Social Networks Use Disorder” (Andreassen & Pallesen, 2014, Brand et al., 2016, Montag et al., 2019). The present study focuses on the use of the SN Facebook and therefore uses the term “Facebook Use Disorder” when referring to the overuse of Facebook. The term “SN Use Disorder” will be used when referring to overuse of SNs in general. We consciously use the term “Disorder” to distinguish disordered use of SNs (involving the symptoms and negative consequences outlined below) from frequent, but non-problematic, use of SNs (see also the debate surrounding the term Internet Use Disorder (e.g. Pontes, Kuss, & Griffiths, 2015)). We view putative SN/Facebook Use Disorder as the extreme pole of a continuum, ranging from no/normal via problematic to disordered use of SNs/Facebook.

Drawing on classifications of substance-use disorders, SN Use Disorder is typically discussed in terms of symptoms such as salience, tolerance, mood modification, withdrawal, conflict, and relapse, as well as problems and negative consequences in daily life (Andreassen, 2015, Griffiths, Kuss, & Demetrovics, 2014). It should be noted, however, that this adaptation of the term and symptoms of addiction to SN use is a topic of debate among researchers, e.g. whether it is appropriate to apply traditional criteria for classifying substance-use disorders to addictive behaviours, such as SN Use Disorder (Kardefelt-Winther et al., 2017).

In addition to these symptoms, the identification of factors that may signal a vulnerability to the development of SN Use Disorders is of interest to researchers and clinicians. Several models and explanations of specific Internet Use Disorders exist (Andreassen, 2015, Brand et al., 2016, Davis, 2001). One prominent model is the Interaction of Person-Affect-Cognition-Execution (I-PACE) Model (Brand et al., 2016). This model proposes the interaction of personal (P), affective (A), cognitive (C), and executive (E) variables in the emergence of a specific Internet Use Disorder. Personality is one of the personal (P) factors implicated in the model and works to create a vulnerability or resilience to the development of a specific Internet Use Disorder (Brand et al., 2016). Drawing on the theoretical framework of this model, the second aim of the present study was to investigate which specific personality traits would be associated with tendencies towards Facebook Use Disorder.

In the field of personality research, one of the most prominent personality models is the Five-Factor Model of personality. According to this model (e.g. (Fiske, 1949)), personality can be described on the basis of five broad factors (commonly referred to as the Big Five). These factors are typically identified as extraversion (being socially outgoing, energetic, vigorous), agreeableness (being helpful, forgiving, unselfish, considerate), conscientiousness (being thorough, orderly, not lazy, efficient), neuroticism (being downcast, tense, emotionally volatile, moody), and openness to experience (being imaginative, interested in arts, aesthetics, new ideas). Depending on the self-report measure used, these five broad factors can be further sub-divided into more precise facets via subscales. For example, the Big Five Inventory (BFI) splits each of the broad Big Five factors into two narrower sub-facets (Rammstedt & Danner, 2017). As such, extraversion is split into the two sub-facets assertiveness and activity. Assertiveness assesses how outgoing and talkative an individual is, whereas activity relates to how energetic and enthusiastic an individual is (Rammstedt and Danner, 2017, Soto and John, 2009).

### 1.2 Previous literature

#### 1.2.1 Users versus non-users of social networks

Putative differences in the Big Five between users and non-users of Facebook and other SNs have previously been investigated. Ryan and Xenos (2011) observed that Facebook users in Australia tended to report higher levels of extraversion but lower levels of conscientiousness relative to non-users. Similarly, Swiss university students who reported higher levels of extraversion and lower levels of conscientiousness were more likely to be a member of a specific SN (Wehrli, 2008). Work by Brailovskaia and Margraf (2016) compared Facebook users and non-users in a sample of German students, but could only replicate the relationship between Facebook use and higher extraversion scores. No relationship was evident between Facebook use and conscientiousness (Brailovskaia & Margraf, 2016). This null result is also at odds with more recent work by Eşkisu, Hoşoğlu, and Rasmussen (2017), who report lower levels of conscientiousness in Facebook users compared to non-users in a sample of Turkish students. Interestingly, in this study, no significant relationship was reported between Facebook use and extraversion, contradicting previous findings (Eşkisu et al., 2017).

One possible explanation for the inconsistent findings in this literature may be related to the fact that none of these studies investigated sub-facets of the Big Five. It is possible that the lack of consideration of the relationship between SN user status and facet-level traits may have resulted in suppressor effects. Such suppressor effects may account for some of the non-significant findings and, therefore, inconsistencies in the results across studies. Moreover, two of the studies used short versions of Big Five measures. Due to the brevity of such measures, it is plausible that the five factors were not fully assessed, which may also explain inconsistent findings between the studies. Finally, samples from different studies also differ in their socio-demographic makeup, e.g. mean age, male-to-female-ratio, and educational background. These differences may also, at least in part, explain the lack of consistency across the results. Despite the existence of such differences, studies rarely report or control for the possible effects of such variables. This lack of consideration of covariates underscores the need to better characterise the populations under study and to control for potential confounding effects of socio-demographic variables.

#### 1.2.2 Social Networks Use Disorder

Extant research considering the association between the broad domains of the Big Five and putative SN Use Disorder has yielded mixed findings. Wilson, Fornasier, and White (2010) found that tendencies toward SN Use Disorder were significantly negatively related to conscientiousness among a sample of Australian students, whereas a significant positive association existed with extraversion. Among a U.S. sample, significant positive correlations were found between neuroticism and a scale assessing Facebook Use Disorder (Blackwell, Leaman, Tramposch, Osborne, & Liss, 2017). In contrast to the work of Wilson et al. (2010), no significant association was observed for extraversion. Notably, agreeableness, conscientiousness, and openness were not investigated in this study (Blackwell et al., 2017). Further work investigating a Taiwanese sample, comprised predominantly of college students, revealed that agreeableness, conscientiousness, and neuroticism were significantly negatively correlated with tendencies towards Facebook Use Disorder (Tang, Chen, Yang, Chung, & Lee, 2016). This latter association stands in contrast to most of the previously mentioned findings. Most recently, a cross-national meta-analysis of findings in the area suggested that Facebook Use Disorder was positively associated with neuroticism and negatively with conscientiousness (Marino, Gini, Vieno, & Spada, 2018). The findings of this meta-analysis compliment the broader literature on addiction and addictive behaviours, which often implicates (low) conscientiousness and (high) neuroticism as vulnerability factors to the development of, e.g. substance addictions (Malouff et al., 2007, Terracciano et al., 2008), problematic Internet use (Kayiş et al., 2016, Lachmann et al., 2019, Montag et al., 2010), and problematic smartphone use (Lachmann et al., 2019).

However, to our knowledge, no previous studies in the field of SN Use Disorder and personality have considered sub-facets of the Big Five, which would help to better characterize the association of personality and SN Use Disorder and might explain some of the heterogeneous findings across studies. The potential of socio-demographic variables such as age, male-to-female-ratio, and educational background, influencing results and potentially explaining inconsistent findings, has also rarely been studied in this context.

### 1.3 Aims, hypotheses, and research questions

The present study followed two major aims: The first aim of the present study was to investigate differences between users and non-users of Facebook with regard to the Big Five personality traits and their sub-facets, while taking into account potential covariates such as age, gender, and education. Secondly, we aimed to investigate the associations of the Big Five and their sub-facets with tendencies towards Facebook Use Disorder. Tests of this aim should again be controlled for the potential covariates, age, gender, and education because these are frequently overlooked in the literature and might help to reconcile some of the inconsistent findings outlined above.

Based on the literature mentioned above, one could hypothesize that (i) Facebook users would score higher in extraversion and lower in conscientiousness compared to Facebook non-users, and that (ii) greater tendencies towards Facebook Use Disorder would be negatively associated with conscientiousness and positively with neuroticism. However, especially with regard to the sub-facets of the Big Five, the present study is of exploratory character. Nevertheless, we deem it as important research questions, to investigate the associations between Facebook use (versus non-use) as well as Facebook Use Disorder and the Big Five sub-facets given the reasons mentioned above.

## 2 Materials and methods

### 2.1 Participants

The study was conducted online via the SurveyCoder tool (https://www.surveycoder.com/; https://www.ckannen.com/). We recruited participants via advertisements placed in newspapers, online, on TV, and over the radio. Whenever a researcher from our group gave an interview on smartphone, Internet, or social media use, the link to the study was presented. Participation was open to anyone who could read and write in German. Participants received feedback on their smartphone use and personality scores. This individualised feedback was used to incentivise participation in the study. Further information about the sampling procedure, exclusion criteria, and handling of outliers can be found in the Supplementary Material. After the exclusion of 60 participants, data from *N* = 3,835 German-speaking participants (2,366 males; 1,469 females) were analysed. The mean age of the sample was *M* = 32.18 (*SD* = 11.82) years. Ages ranged from 11 to 75 years. Of the total *N* = 3,835 participants, 2,629 (1,577 males; 1,052 females) reported having a Facebook account. Thus, 1,206 participants (789 males; 417 females) reported that they did not have a Facebook account.

### 2.2 Ethical approval and informed consent

The study was approved by the local ethics committee of Ulm University, Ulm, Germany. Informed consent was obtained electronically from all participants prior to their participation in the study. Underaged participants were asked to obtain consent from their parents/legal guardians before participating.

### 2.3 Self-report measures

#### 2.3.1 Demographics

Participants completed demographic questions, including their age, gender, and their highest level of education.

#### 2.3.2 Big Five Inventory (BFI)

Participants also completed the German version of the Big Five Inventory (BFI) (Rammstedt & Danner, 2017). This questionnaire comprises 45 items, answered on a 5-point Likert-Scale, which ranges from 1 = “very inapplicable” to 5 = “very applicable”. The German version of the BFI contains one additional item for the agreeableness scale compared to the English language version of this questionnaire. To enable closer comparison with the English version of the measure, we omitted this additional item from our analyses. The mean scores of the five broad factors, as well as two sub-facets/subscales for each of the broad factors were calculated for each participant. The internal consistencies (assessed using Cronbach’s α) for the Big Five factors were: extraversion: α = 0.86, agreeableness: α = 0.72, conscientiousness: α = 0.82, neuroticism: α = 0.85, and openness: α = 0.79. The internal consistencies for the subscales were: extraversion: assertiveness: α = 0.83, activity: α = 0.59; agreeableness: altruism: α = 0.58, compliance: α = 0.44; conscientiousness: order: α = 0.65, self-discipline: α = 0.69; neuroticism: anxiety: α = 0.76, depression: α = 0.57; openness: aesthetics: α = 0.79, ideas: α = 0.58. Although several of the internal consistencies seem rather low, this can be accounted for by the low number of items per subscale (e.g. there are only three items on the compliance scale, which likely explains the relatively low α = 0.44). The present values also closely reflect those reported by Rammstedt and Danner (2017). Thus, we deemed the internal consistencies acceptable for the present sample. Additional information on the mean inter-item correlations for each subscale can be found in the Supplementary Material.

#### 2.3.3 Facebook use and tendencies towards Facebook Use Disorder

Finally, participants answered yes/no to the question: “Do you have a Facebook account?” (answer options: “yes” (=Facebook users) and “no” (=Facebook non-users)). It was not assessed whether participants had ever had a Facebook account (i.e. and subsequently deleted it). If participants indicated that they had a Facebook account, they were asked to complete a 10-item Facebook Use Disorder scale (FUD-S). This scale was developed by our research team, based on the short version of the smartphone addiction scale (Kwon, Kim, Cho, & Yang, 2013). Thus, the term “smartphone” was replaced by “Facebook” for each item to reflect the putative Facebook Use Disorder. Based on previous work, we also reworded a small number of items to reflect the first-person perspective to make the items more clear for participants (Duke & Montag, 2017). Items were answered on a 6-point Likert-Scale ranging from 1 = “strongly disagree” to 6 = “strongly agree”. This questionnaire has previously been used and validated using a Confirmatory Factor Analysis by Sha, Sariyska, Riedl, Lachmann, and Montag (2019). A mean score across the 10 items was calculated for each participant. The internal consistency of the FUD-S was α = 0.96 in the present sample. Further information on the fit of the FUD-S in the present sample is presented in the Supplementary Material.

### 2.4 Statistical analyses

Analyses were conducted using the statistical software, R (Version 3.4.1 (R core team, 2017)), and R-Studio (Version 1.1.463 (RStudio Team, 2015)).

Information on the distributions of the BFI (sub-)scales and the FUD-S, as well as the associations of these variables with age, gender, and educational background, can be found in the Supplementary Material. These results prompted us to control for age and gender (but not educational background) in subsequent analyses.

To investigate between group differences in personality between Facebook users and non-users, a multifactorial multivariate ANCOVA and separate multifactorial ANCOVAs were calculated. Gender and Facebook use were entered as independent variables, age was included as a covariate. To correct for multiple comparisons, the alpha level was set to α = 0.05/15 = 0.0033, as a total of 15 (sub-)scales of the BFI were examined.

To investigate associations between the BFI (sub-)scales and Facebook Use Disorder, Spearman partial correlations (corrected for age) between the BFI (sub-)scales and the scores in the FUD-S were calculated for the complete, male, and female sample of Facebook users. For these analyses, the alpha level was also set to α = 0.05/15 = 0.0033.

As we did not have a directional hypothesis for each association under investigation, we tested all associations for significance in a two-tailed manner. The analyses (alongside the data) are also registered at the open science framework (https://osf.io/qf2cu/).

## 3 Results

### 3.1 Differences in personality between users and non-users of Facebook

A multifactorial multivariate ANCOVA and separate multifactorial ANCOVAs, with gender and Facebook use as independent variables and age as covariate, revealed significant (*p* < 0.05) differences between males and females for nearly all (sub-)scales of the BFI. Only the agreeableness subscale compliance yielded non-significant findings. Females scored higher than males on all (sub-)scales, except the ideas subscale of the factor openness (males > females). All significant results survived manual adjustment for multiple comparisons (α = 0.0033). However, most effect sizes were small. Please see Table 1 for descriptive statistics and detailed results.

**Table 1:** Descriptive statistics for the BFI for the complete sample, for the sample split by gender and the results of multifactorial ANCOVAs (effects of gender as independent variable).

	Complete sample (*N* = 3,835)		Males (*n* = 2,366)		Females (*n* = 1,469)		Results ANCOVAs		
	*M*	*SD*	*M*	*SD*	*M*	*SD*	*F*(1, 3830)	*p*	η_p_^2^
**Extraversion**	3.31	0.77	3.23	0.77	3.44	0.76	60.31	<0.001	0.016
Assertiveness	3.25	0.87	3.17	0.87	3.38	0.86	50.86	<0.001	0.013
Activity	3.45	0.80	3.38	0.80	3.57	0.78	44.97	<0.001	0.012
**Agreeableness**	3.47	0.55	3.42	0.54	3.54	0.56	42.18	<0.001	0.011
Altruism	3.49	0.64	3.41	0.62	3.62	0.64	83.32	<0.001	0.021
Compliance	3.47	0.70	3.47	0.68	3.49	0.72	2.30	0.130	0.001
**Conscientiousness**	3.40	0.64	3.33	0.64	3.51	0.63	84.48	<0.001	0.022
Order	3.09	1.01	3.01	1.00	3.21	1.02	43.37	<0.001	0.011
Self-discipline	3.38	0.65	3.32	0.64	3.47	0.65	62.65	<0.001	0.016
**Neuroticism**	2.84	0.76	2.72	0.74	3.05	0.75	111.01	<0.001	0.028
Anxiety	2.92	0.85	2.77	0.82	3.18	0.83	141.76	<0.001	0.036
Depression	2.69	0.95	2.62	0.95	2.81	0.94	21.03	<0.001	0.005
**Openness**	3.65	0.60	3.63	0.60	3.69	0.60	11.97	<0.001	0.003
Aesthetics	3.52	1.00	3.39	1.00	3.72	0.95	101.04	<0.001	0.026
Ideas	3.71	0.58	3.74	0.58	3.65	0.58	18.58	<0.001	0.005

*Note.* Scales assessing the broad Big Five factors are bolded. η_p_^2^ = partial eta squared.

**Fig. 1:**
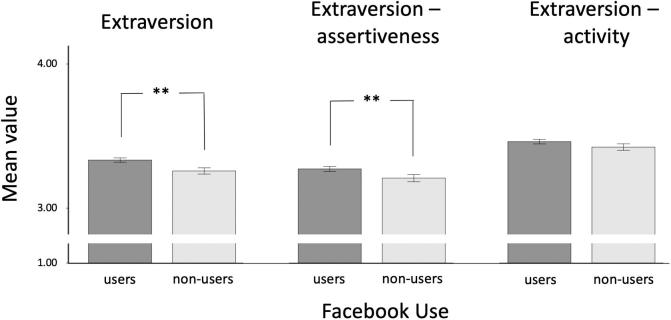
Differences between Facebook users and non-users in extraversion and its subscales (*M* +/− 2 *SE*). ***p < 0.001, **p < 0.01, *p < 0.05, two-tailed. Please note that the possible range of the BFI (sub-)scales is 1 to 5.

**Fig. 2:**
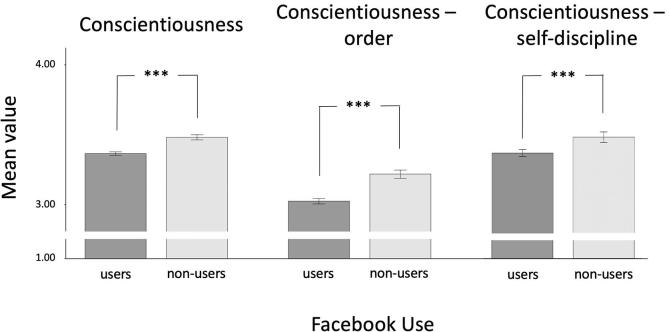
Differences between Facebook users and non-users in conscientiousness and its subscales (*M* +/− 2 *SE*). ***p < 0.001, **p < 0.01, *p < 0.05, two-tailed. Please note that the possible range of the BFI (sub-)scales is 1 to 5.

The multifactorial multivariate ANCOVA and separate multifactorial ANCOVAs also revealed significant (*p* < 0.05) differences between Facebook users and non-users on several (sub-)scales of the BFI. However, when considering the manually adjusted alpha-level of 0.0033, only the results for the complete extraversion scale (users > non-users) and for conscientiousness and all its subscales (users < non-users) remained significant. Overall, effect sizes were small. Please see Table 2 for descriptive statistics and more detailed results. Fig. 1, Fig. 2 provide a graphical illustration of these results.

**Table 2:** Descriptive statistics for the BFI for users and non-users of Facebook and the results of multifactorial ANCOVAs (effects of user group as independent variable).

	Users (*n* = 2,629)		Non-users (*n* = 1,206)		Results ANCOVAs		
	*M*	*SD*	*M*	*SD*	*F*(1, 3830)	*p*	η_p_^2^
**Extraversion**	3.33	0.78	3.26	0.76	9.64	0.002	0.003
Assertiveness	3.27	0.88	3.21	0.86	7.02	0.008	0.002
Activity	3.46	0.80	3.42	0.79	1.50	0.220	0.000
**Agreeableness**	3.48	0.56	3.44	0.53	4.67	0.031	0.001
Altruism	3.51	0.64	3.45	0.62	6.09	0.014	0.002
Compliance	3.48	0.70	3.47	0.71	0.00	0.999	0.000
**Conscientiousness**	3.36	0.64	3.48	0.64	16.76	<0.001	0.004
Order	3.02	1.01	3.22	1.02	24.15	<0.001	0.006
Self-discipline	3.34	0.64	3.45	0.65	11.50	<0.001	0.003
**Neuroticism**	2.88	0.77	2.77	0.74	7.12	0.008	0.002
Anxiety	2.96	0.86	2.84	0.83	7.89	0.005	0.002
Depression	2.72	0.95	2.62	0.93	2.11	0.146	0.001
**Openness**	3.65	0.60	3.66	0.59	0.01	0.941	0.000
Aesthetics	3.51	1.01	3.54	0.98	0.06	0.809	0.000
Ideas	3.70	0.58	3.72	0.58	0.04	0.844	0.000

*Note.* Scales assessing the broad Big Five factors are bolded. η_p_^2^ = partial eta squared.

The multivariate effect of the interaction between Facebook use and gender on BFI scores was not significant (*F*(15, 3816) = 1.43, *p* = 0.126). Therefore, the interaction effects on each BFI (sub-)scale were not investigated further.

### 3.2 Correlations between tendencies towards Facebook Use Disorder and personality

The mean values on the FUD-S for the Facebook users sample ranged from 1 to 6 with a median of 1.30 and a mean value of *M* = 1.62 (*SD* = 0.76). As outlined in Table 3, several significant partial (corrected for age) Spearman correlations were found between the BFI (sub-)scales and the FUD-S score in the group of Facebook users (*n* = 2,629). The strongest effects in the complete users sample, which survived Bonferroni correction for multiple comparisons (α = 0.005/15 = 0.0033), were observed between the FUD-S and: compliance, a sub-facet of agreeableness (*r_s_* = −0.11, *p* < 0.001); conscientiousness (*r_s_* = −0.12, *p* < 0.001) and its subscale self-discipline (*r_s_* = −0.15, *p* < 0.001); neuroticism (*r_s_* = 0.17, *p* < 0.001) and its subscales, anxiety (*r_s_* = 0.15, *p* < 0.001) and depression (*r_s_* = 0.17, *p* = 0.001); and the ideas subscale of openness (*r_s_* = −0.07, *p* < 0.001).

**Table 3:** Partial Spearman correlations between the BFI and the Facebook Use Disorder Scale for the complete sample of Facebook users and split by gender.

	All users (*n* = 2,629)	Male users (*n* = 1,577)	Female users (*n* = 1,052)
**Extraversion**	*r_s_* = 0.04	*r_s_* = 0.02	*r_s_* = 0.03
	*p* = 0.054	*p* = 0.455	*p* = 0.263
Assertiveness	*r_s_* = 0.05	*r_s_* = 0.03	*r_s_* = 0.05
	*p* = 0.017	*p* = 0.288	*p* = 0.140
Activity	*r_s_* = −0.02	*r_s_* = −0.03	*r_s_* = −0.05
	*p* = 0.236	*p* = 0.317	*p* = 0.126
**Agreeableness**	*r_s_* = −0.04	*r_s_* = −0.07	*r_s_* = −0.04
	*p* = 0.028	*p* = 0.005	*p* = 0.240
Altruism	*r_s_* = 0.02	*r_s_* = 0.00	*r_s_* = 0.01
	*p* = 0.238	*p* = 0.914	*p* = 0.702
Compliance	*r_s_* = −0.11	*r_s_* = −0.13	*r_s_* = −0.09
	*p* < 0.001	*p* < 0.001	*p* = 0.002
**Conscientiousness**	*r_s_* = −0.12	*r_s_* = −0.12	*r_s_* = −0.17
	*p* < 0.001	*p* < 0.001	*p* < 0.001
Order	*r_s_* = −0.06	*r_s_* = −0.04	*r_s_* = −0.10
	*p* = 0.003	*p* = 0.076	*p* < 0.001
Self-discipline	*r_s_* = −0.15	*r_s_* = −0.14	*r_s_* = −0.19
	*p* < 0.001	*p* < 0.001	*p* < 0.001
**Neuroticism**	*r_s_* = 0.17	*r_s_* = 0.17	*r_s_* = 0.14
	*p* < 0.001	*p* < 0.001	*p* < 0.001
Anxiety	*r_s_* = 0.15	*r_s_*=0.14	*r_s_* = 0.11
	*p* < 0.001	*p* < 0.001	p < 0.001
Depression	*r_s_* = 0.17	*r_s_* = 0.17	*r_s_* = 0.15
	*p* < 0.001	*p* < 0.001	*p* < 0.001
**Openness**	*r_s_* = −0.04	*r_s_* = −0.06	*r_s_* = −0.02
	*p* = 0.045	*p* = 0.013	*p* = 0.622
Aesthetics	*r_s_* = 0.02	*r_s_* = 0.00	*r_s_* = −0.01
	*p* = 0.379	*p* = 0.849	*p* = 0.722
Ideas	*r_s_* = −0.07	*r_s_* = −0.09	*r_s_* = −0.03
	*p* < 0.001	*p* < 0.001	*p* = 0.415

*Note.* Scales assessing the broad Big Five factors are bolded. This table presents data for the Facebook users sample (*n* = 2,629), only. All correlations are corrected for age. *p*-values are derived from two-tailed tests.

Similar patterns of correlations were found for both males and females after splitting the sample by gender. Exceptions to this pattern were: (1) the relationship between order, a subscale of conscientiousness, and the FUD-S, which remained significant only among females, after correcting for multiple comparisons (*r_s_* = −0.10, *p* < 0.001); and (2) the correlation between the openness subscale, ideas, and the FUD-S, which was not significant for the female sub-sample. However, this latter correlation did not differ significantly between males and females (*z* = 1.56, *p* = 0.119; *p*-values for all other differences in the correlations between males and females > 0.143).

## 4 Discussion

The first aim of the present study was to investigate differences between users and non-users of Facebook with regard to the Big Five personality traits, while taking into account potential covariates such as age and gender (and education). Secondly, we aimed to investigate the associations between the Big Five and tendencies towards Facebook Use Disorder. Tests of these aims again controlled for the potential covariates, age and gender (and education). Finally, all analyses included exploratory analyses of possible associations between Facebook use, Facebook Use Disorder and sub-facets of the Big Five, to better characterise the relationships between personality and Facebook use.

### 4.1 Differences between Facebook users and non-users in personality

We found that our sample of Facebook users was typically younger, showed a lower male-to-female-ratio, and had a higher educational level, relative to non-users (see Supplementary Material). Additionally, we replicated previous findings that Facebook users score higher in extraversion, but lower in conscientiousness (Brailovskaia and Margraf, 2016, Eşkisu et al., 2017, Ryan and Xenos, 2011, Wehrli, 2008), however, these findings should be interpreted with caution, given the relatively small effect sizes observed for the present sample. Nevertheless, the direction of effects is in line with our previous assumptions and, in contrast to previous studies, these results controlled for the potential confounding effects of age and gender.

Interestingly, sub-facets of extraversion were differentially related to the use (or non-use) of Facebook. The assertiveness facet differed significantly between Facebook users and non-users (η_p_^2^ = 0.002), but the activity facet did not (η_p_^2^ < 0.001). This finding suggests that those individuals who particularly like to communicate with others are more likely to use Facebook (Rammstedt & Danner, 2017). At this point it is important to note that the assertiveness facet of extraversion includes items such as “I see myself as someone who is talkative”, “I see myself as someone who is reserved” (latter one reversed scored) (Rammstedt & Danner, 2017). Hence, the present results support previous work by Kujath (2011) and Raacke and Bonds-Raacke (2008), who argue that staying in touch with friends and acquaintances is one motivation for using SNs. Accordingly, as Kujath (2011) concludes, the use of SNs like Facebook may be seen as an extension of offline social interactions. We urge caution in this interpretation, however, as only the user-group difference for extraversion as a whole – not for the individual sub-facets – survived the correction for multiple comparisons in the current sample. Regarding conscientiousness, Facebook non-users scored higher on all conscientiousness (sub-)scales and these effects remained significant after Bonferroni correction. Larger effects were found for the order facet compared to self-discipline (η_p_^2^ = 0.006 vs. η_p_^2^ = 0.003). This suggests that, relative to Facebook users, non-users are more orderly, carry out their jobs and duties more carefully and reliably (Rammstedt & Danner, 2017). One possible explanation for these differences is that conscientious individuals want to prevent the possibility of SNs negatively affecting their productivity, e.g. at work or school. Therefore, they may prefer not to use SNs like Facebook. This interpretation is supported by earlier work, suggesting that SNs were viewed as too time consuming by teenagers who chose not to use them (Baker & White, 2010).

Despite the relatively small effect sizes observed, these results have important implications for future research. An increasing amount of research is conducted via SNs like Facebook and dedicated online research participation platforms. The socio-demographic and personality differences between users and non-users of Facebook highlighted by the present study indicate that samples drawn from the population of Facebook users may not be representative of the general population, which also includes non-users of Facebook. Hence, the generalisability of results derived from samples of Facebook-users is questionable. Consideration of these differences is particularly important given the growing number of studies aiming to investigate digital footprint data from SNs via Psychoinformatics methods (Insel, 2017, Montag et al., 2016). Finally, although the effect sizes observed for the differences between users and non-users of Facebook are rather small in the present study, knowledge about the specific characteristics of Facebook users is heavily monetised. This is particularly important given the increased interest in mass persuasion (e.g. personalised advertising) tailored to personality information gleaned from digital footprints (Matz, Kosinski, Nave, & Stillwell, 2017). Given the vast popularity of Facebook (We Are Social, Hootsuite, & DataReportal, 2019), even such small effects may have important implications. Thus, the present findings are important in refining our understanding of the populations targeted by such approaches.

### 4.2 Associations between Facebook Use Disorder and personality

As well as characterising differences between users and non-users of Facebook, conscientiousness was also significantly negatively related to tendencies towards Facebook Use Disorder within the users sample. This is in line with previous literature (e.g. Tang et al., 2016, Wilson et al., 2010). Moreover, this association was found for males as well as females, and all results were controlled for the potential confounding effect of age. This relationship suggests that even among those who use SNs like Facebook, individuals who are more conscientious are less likely to develop tendencies towards SN Use Disorder, such as Facebook Use Disorder. Interestingly, this relationship was characterised by a stronger association with the subscale self-discipline in comparison to the subscale order (*r_s_* = −0.15 vs. *r_s_* = −0.06). As noted previously, it may be that more conscientious individuals realise that SN use may negatively influence their productivity. The relationship between tendencies toward Facebook Use Disorder and scores on the self-discipline subscale may indicate that individuals who score highly on this subscale are proficient and potentially do not want to waste time on SNs (Baker & White, 2010). Thus, such individuals may be more skilled at controlling the amount of time spent on SNs like Facebook, and in preventing possible negative influences of SNs on their lives. However, again, the effect sizes found in the present study are relatively small and thus caution should be observed when interpreting these findings.

Neuroticism and its subscales, depression and anxiety, were positively related to tendencies towards Facebook Use Disorder. These findings remained significant after correction for multiple comparisons, among both males and females. Again, the results were controlled for the potential confounding effect of age. As the correlations with the Facebook Use Disorder scale were similarly sized for both facets of neuroticism, we cannot offer a more nuanced interpretation of the relationship between neuroticism and tendencies towards Facebook Use Disorder. Interestingly, a recent study by Peterka-Bonetta, Sindermann, Sha, Zhou, and Montag (2019) reported a relationship, in which depressive symptoms (measured with the Beck Depression Inventory – II (Beck, Steer, & Brown, 1996)) were significantly positively associated with tendencies towards Internet Use Disorder. These results underline the importance of the present findings with respect to the depression subscale of neuroticism.

As mentioned in the introduction, both (higher) neuroticism and (lower) conscientiousness are also predictors of other kinds of addictions and addictive behaviours, such as (unspecified) Internet Use Disorder (Kayiş et al., 2016, Lachmann et al., 2019, Malouff et al., 2007, Montag et al., 2010, Montag et al., 2011, Terracciano et al., 2008). Therefore, the present study lends some support to the interpretation of Facebook Use Disorder as having an addictive nature and suggests it may be viewed as a specific Internet Use Disorder. The putatively addictive nature of Facebook has also been investigated in the realm of app-design: Certain elements of Facebook and other social media platforms seem to have been designed to prolong online time and to elicit fear of missing out (Montag, Lachmann et al., 2019). These arguments also strengthen the necessity to discuss nomenclature and symptoms related to a potential diagnosis of SN Use Disorder in future revisions of the International Classification of Diseases and the Diagnostic and Statistical Manual of Mental Disorders. However, more evidence is necessary to inform this discussion. It is also important not to over-interpret the present findings, as the effect sizes of the correlations with neuroticism and conscientiousness were quite small and direct comparisons with other addictive behaviours were not made.

Finally, the negative associations of Facebook Use Disorder with compliance (a subscale of agreeableness) and ideas (a subscale of openness) survived Bonferroni correction for multiple testing. These results suggest that participants who criticise others and tend to argue with others (low scores in the compliance subscale (Rammstedt & Danner, 2017)) show higher tendencies towards Facebook Use Disorder. Potentially, social interactions involving disagreements are easier to handle in online, relative to offline, environments. This relative ease of interaction may in turn reinforce the use of SNs like Facebook, ultimately leading to higher tendencies towards the disordered use. The association with the ideas facet of openness suggests that people who are interested in different topics and new experiences (high scores in the ideas subscale (Rammstedt & Danner, 2017)) show lower tendencies towards Facebook Use Disorder. This association might be explained by the fact that individuals scoring high in the subscale ideas might strive for new experiences offline rather than spending a lot of time in front of a computer/smartphone on SNs. Alternatively, work by Marshall, Lefinghausen and Ferenczi (2015) suggests that people higher in openness may have different motivations for using Facebook, e.g. for sharing information on intellectual topics, rather than purely social interactions, which may facilitate more goal-directed engagement with the SN. However, these interpretations are preliminary and the findings reported here need further investigation and replication.

### 4.3 Limitations

Some potential shortcomings of the present study should also be discussed. First, the generalisability of the present results may be limited to Facebook. Facebook is clearly one of the largest SNs (We Are Social, Hootsuite, & DataReportal, 2019) and is the most frequently investigated SN, as highlighted in the introduction to this paper. Recent work by Marshall, Ferenczi, Lefringhausen, Hill, and Deng (2020) points to personality differences between Facebook users and Twitter users, suggesting Twitter users are higher in both openness and Machiavellianism. Thus, it is advisible to replicate the present study and to consider if different personality profiles predict engagement with different SN platforms. Secondly, FUD-S scores were quite low for most of the participants in this sample (as indicated by a median of 1.30, and a mean of *M* = 1.62 (*SD* = 0.76)). This limits the variance in the data and ultimately the generalisability of these results to samples showing more severe symptoms of SN/Facebook Use Disorder. These low scores may also explain some of the rather small effect sizes found in the present study. Next, the generalisability of the present results to samples from other cultures is questionable. Our findings broadly reflect those from an Australian sample of Internet users (Ryan & Xenos, 2011). Thus, it is likely that these findings at least generalise to Western cultures. Similarly, it should be mentioned that inconsistent findings in previous studies could be due to cultural differences in the samples, as well as measurement variance in the measures used in different countries (Błachnio, Przepiorka, Senol-Durak, Durak, & Sherstyuk, 2017). Unfortunately, it was not possible to test the moderating effect of country within the present dataset and this should be a goal for future work. Next, the present sample may be biased towards individuals who are interested in smartphones and/or social media, as much of the recruitment was carried out via media related to these topics, e.g. the study was advertised whenever one of the researchers from our group gave an interview on this topic. Crucially, the variable Facebook user status did not differentiate between participants who never had a Facebook account and participants who had one in the past, but have since deleted it. It is possible that different personality characteristics also exist between these two groups. Another possible limitation is that no causal direction can be inferred based on the present data, due to the cross-sectional design used in the study. However, the Big Five are understood as rather robust and stable personality factors, which manifest in specific behavioural patterns (Costa & McCrae, 1992). Hence, it is likely that differences in the Big Five causally explain whether individuals use Facebook or not and why some individuals may develop tendencies towards Facebook Use Disorder. As mentioned previously, the I-PACE model proposes that personality factors, including low conscientiousness, are variables that may predispose individuals to develop and maintain specific kinds of Internet Use Disorders (Brand et al., 2016). As we view Facebook Use Disorder as a specific form of Internet Use Disorder, this model holds explanatory power for the present data.

### 4.4 Future work

Future work should consider which specific functions of Facebook are driving the use of Facebook and other SNs. For example, it remains unclear whether high extraversion is associated with Facebook use solely because of the platform’s communication functionality (Kujath, 2011, Raacke and Bonds-Raacke, 2008), or if this relationship can also be explained via other Facebook features. The present data do not permit conclusions as to whether tendencies towards Facebook Use Disorder pertain to overall Facebook use or towards certain functions of Facebook. This is underlined by work by Rothen et al. (2018), who found that problematic use of Facebook was linked to preferences for specific functions. Hence, it is also unclear whether high neuroticism is linked to addictive tendencies towards all functions of Facebook or only toward specific functions. A meta-analysis by Liu and Campbell (2017) suggested that the Big Five show differential associations with the use (not tendencies towards a Use Disorder) of specific SN activities. For example, neuroticism was significantly associated with updating one’s status, but not with SN gaming, information seeking, interaction or any of the other specific activities investigated (Liu & Campbell, 2017). Hence, analysing associations between the Big Five (and their sub-facets) and specific activities carried out via the SN and/or sub-types of disordered SN use, would be an interesting avenue for future research.

Finally, personality is not the only variable of interest when investigating Facebook use versus non-use or Facebook Use Disorder. Research indicates the existence of individual differences between groups of Facebook users, not limited to differences in personality. This indicates a complex interplay of many variables, which influence how individuals use Facebook. Most likely, such groups are also differentially susceptible to the development of a Facebook Use Disorder (Lo Coco et al., 2018, Moreau et al., 2015).

## 5 Conclusions

The present study builds on the existing literature by providing evidence that Facebook users report higher extraversion and lower conscientiousness scores than non-users in a large, German-speaking sample. Facebook users also differed from non-users in key socio-demographic variables, including age and male-to-female-ratio. This has crucial implications for future studies aiming to collect data from Facebook users. Additionally, we provide evidence that low conscientiousness and high neuroticism are linked to tendencies towards Facebook Use Disorder. Therefore, the present study provides further nuance to our understanding of the associations of socio-demographic variables, personality, and Facebook use.

## Funding

This work was supported by the German Academic Scholarship Foundation (Studienstiftung des deutschen Volkes) [CS did hold a scholarship from this foundation]; the German Research Foundation (Deutsche Forschungsgemeinschaft) [grant number DFG, MO2363/3-2]. None of the funding sources had any influence on the study design; the collection, analysis and interpretation of the data; on writing of the manuscript; or on the decision to submit the manuscript for publication.

Authors’ contributions

CM planned the study design and implemented data collection. CS conducted the statistical analyses, writing of the manuscript and interpretation of the data. CM worked over the manuscript. ÉD also worked over the manuscript, gave helpful advice and improved the English language. All authors agreed upon the final version and submission of the manuscript.

## CRediT authorship contribution statement

**Cornelia Sindermann:** Data curation, Formal analysis, Methodology, Writing - original draft, Writing - review & editing. **Éilish Duke:** Writing - review & editing. **Christian Montag:** Data curation, Methodology, Project administration, Resources, Validation, Writing - review & editing.

## Declarations of Competing Interest

Dr. Montag mentions that he has received (to Ulm University and earlier University of Bonn) grants from the German Research Foundation (DFG) and the German Federal Ministry for Research and Education. Dr. Montag has performed grant reviews for several agencies; has edited journal sections and articles; has given academic lectures in clinical or scientific venues or companies; and has generated books or book chapters for publishers of mental health texts. For some of these activities he received royalties, but never from the gaming or social media industry. Dr. Montag mentions that he is part of a discussion circle (Digitalität und Verantwortung: https://about.fb.com/de/news/h/gespraechskreis-digitalitaet-und-verantwortung/) debating ethical questions linked to social media, digitalization and society/democracy at Facebook. In this context, he receives no salary for his activities. The authors declare that they have no competing interests. Finally, Dr. Montag mentions that he is currently on the scientific advisory board of the Nymphenburg group.
