# Supplementary material for: Personality associations with Facebook use and tendencies towards Facebook Use Disorder
Source: Addict Behav Rep. 2020 Feb 19;11:100264. doi: 10.1016/j.abrep.2020.100264 (PMC7244926; doi:10.1016/j.abrep.2020.100264)
Supplement: Supplementary data 2 [file mmc2.pdf]

Personality associations with Facebook use and tendencies towards Facebook Use Disorder

Sampling Procedure, Exclusion Criteria, and Handling of Outliers

The present online study aimed to assess data from as many German(-speaking) participants as possible. We sought to recruit the most representative German(-speaking) sample possible to study the topic of smartphone and social media use. We aimed for a final sample size of at least N=3,000 participants after excluding those participants who did not meet the inclusion criteria (see also registration of the present study: https://osf.io/qf2cu/).

A total of N=3,895 participants, mostly from Germany, but also from Austria and Switzerland, participated in the present study. Of these, 48 participants were excluded because they did not own a smartphone. This was an exclusion criterion, as only those participants who owned a smartphone were subsequently asked whether they had a Facebook account. This is because the study was designed to also investigate Smartphone Use Disorder. This aim is discussed in a separate paper, which investigates the relationship between WhatsApp, Facebook, and Smartphone Use Disorder and variables assessing Fear of Missing Out and life satisfaction (Sha, Sariyska, Riedl, Lachmann, & Montag, 2019). Please also note that the present sample partly overlaps (n=41) with the sample of Sindermann, Elhai, Moshagen, and Montag (2020). Additionally, participants, who gave incomplete (no participants) and/or implausible answers (e.g. an age of 0 or 1 years or more than 100 years (12 participants)) were excluded. In the end, N=3,835 participants (n=2,366 males, n=1,469 females) were included in the final analyses. No age restrictions were imposed on participants from an ethics perspective. However, we sought to recruit participants over the age of 12 and those participants aged 12-18 were required to provide electronic consent from their parents / legal guardians. Despite this message, two participants aged 11 years participated. As we obtained electronic parental consent, and as no age restriction was required by the ethics committee, these participants were included in the analyses.

Due to similarities in cultural and geographical backgrounds, we collapsed the German (n=3,590), Austrian, and Swiss participants into one large sample. The current sample does not exactly represent the gender distribution of the general German, Austrian or Swiss population. This is approximately 50:50 in all the three countries (see registration at https://osf.io/qf2cu/) (Bundesministerium für Gesundheit und Frauen, 2016; Bundesamt für Statistik as cited in Statista, 2018; Bundesamt für Statistik (BFS), 2016). In contrast, the makeup of the present sample is around 60:40 males to females. However, as we calculated results separately for males and females (see significant differences between gender in a number of variables presented below), we decided to not exclude more participants (to gain a 50:50 male-to-female-ratio).

In the registration documentation pertaining to this project (see online at the Open Science Framework - https://osf.io/qf2cu/) we transparently report how we planned to handle the inclusion or exclusion of outliers. In brief, we did not exclude any outliers for the analyses and results presented in the main manuscript. However, we also implemented all of the analyses with a sub-sample of the participants, which excluded all outliers. All participants scoring lower than [25_{th}-Quantile - (1.5×(75_{th}-Quantile - 25_{th}-Quantile))] or higher than [75_{th}-Quantile + (1.5×(75_{th}-Quantile - 25_{th}-Quantile))] with respect to age, the BFI (sub-)scales, and/or the Facebook Use Disorder scale (FUD-S), were treated as outliers (Tukey, 1977). A sample of N=3,526 (n=2,153 males) remained. The results for this smaller sample are not presented in the main manuscript, as the results remained similar irrespective of whether outliers were included in – or excluded from – the analyses. Only the p-values differed slightly, leading to marginally statistically significant results in the sample without outliers for a small number of cases. Based on these few, and from our perspective, minor differences, it was decided to present the results for the complete sample (i.e. including the outliers) to ensure the highest level of statistical power.

References

Bundesministerium für Gesundheit und Frauen. *Frauen und Männer in Österreich: Gender Index 2016*. 2016. https://www.bmgf.gv.at/cms/home/attachments/6/9/9/CH1553/CMS1465897036085/gender_index_2016.pdf. Accessed 22 August 2018.

Bundesamt für Statistik (BFS). *Die Bevölkerung der Schweiz 2015.* 2016. https://www.bfs.admin.ch/bfsstatic/dam/assets/1401562/master. Accessed 22 August 2018.

Statista (2018). Bevölkerung - *Einwohnerzahl in Deutschland nach Geschlecht von 1995 bis 2017 (in 1.000).* 2019. https://de.statista.com/statistik/daten/studie/161868/umfrage/entwicklung-der-gesamtbevoelkerung-nach-geschlecht-seit-1995/. Accessed 18 March 2019.

Sha, P., Sariyska, R., Riedl, R., Lachmann, B., & Montag, C. (2019). Linking internet communication and smartphone use disorder by taking a closer look at the Facebook and WhatsApp applications. *Addictive behaviors reports, 9*, 100148. https://doi.org/10.1016/j.abrep.2018.100148

Sindermann, C., Elhai, J. D., Moshagen, M., & Montag, C. (2020). Age, gender, personality, ideological attitudes and individual differences in a person's news spectrum: how many and who might be prone to “filter bubbles” and “echo chambers” online?. Heliyon, 6(1), e03214. https://doi.org/10.1016/j.heliyon.2020.e03214

Tukey JW. *Exploratory data analysis. Vol. 2*. Reading, Mass.: Pearson; 1977.

Inter-Item Correlations of the Big Five Inventory Subscales

Supplementary Table 1
Inter-item correlations of the BFI subscales

Subscale	Mean inter-item correlation [minimum; maximum]	Number of items
**Extraversion**		
Assertiveness	.49 [.31; .73]	5
Activity	.41	2
**Agreeableness**		
Altruism	.27 [.14; .39]	4
Compliance	.21 [.15; .32]	3
**Conscientiousness**		
Order	.49	2
Self-discipline	.32 [.26; .38]	5
**Neuroticism**		
Anxiety	.44 [.35; .65]	4
Depression	.40	2
**Openness**		
Aesthetics	.56 [.48; .70]	3
Ideas	.23 [.04; .40]	5

Analyses of the Fit of the Facebook Use Disorder Scale in the Present Sample

A Principal Component Analysis revealed one component with an eigenvalue greater than one (5.73) and also the screeplot pointed towards a one-component solution. A Confirmatory Factor Analysis revealed the following scores: Comparative Fit Index = 0.89, Tucker-Lewis Index = 0.86, Root Mean Squared Error of Approximation = 0.13, Standardised Root Mean Squared Residual = 0.05 and loadings of the items on the one factor lied between .55 and .82. Therefore, we deemed the fit as acceptable, although, not perfect. The mean inter-item correlation was .52 (minimum: .35; maximum: .75) and the item-total correlations ranged from .52 to .76.

Distributions

As tests assessing the normal distribution of the data would likely be biased towards significance due to the large sample size, we visually inspected histograms of all variables to assess their normality. We also examined the skewness and kurtosis values for each variable (see rule of thumbs by Miles and Shevlin (2001)). In comparison to the FUD-S (skewness=1.56, kurtosis=2.44, in N=2,629, who were identified as Facebook users), all scales and subscales of the BFI showed a skewness and a kurtosis of less than +/-1 and the histograms resembled a normal distribution (this was also the case when the sample was split by gender and/or Facebook user status).

Therefore, parametric tests were used for all analyses not including the FUD-S but non-parametric tests were used when the FUD-S was included in the analyses. Please see Supplementary Table 2 and Supplementary Figures 1 and 2 for more details on the distributions.

The data used in the main manuscript / Supplementary Material is also available at https://osf.io/qf2cu/ if one would like to create histograms for certain (sub-)scales or (sub-)samples.

Supplementary Table 2

Range, skewness, and kurtosis of the distributions of all (sub-)scales under investigation in the complete sample

	Min	Max	Skewness	Kurtosis
**Extraversion**	1.00	5.00	-0.15	-0.52
Assertiveness	1.00	5.00	-0.12	-0.68
Activity	1.00	5.00	-0.27	-0.15
**Agreeableness**	1.33	5.00	-0.31	0.17
Altruism	1.00	5.00	-0.40	0.22
Compliance	1.00	5.00	-0.29	-0.15
**Conscientiousness**	1.11	5.00	-0.19	-0.22
Order	1.00	5.00	0.00	-0.83
Self-discipline	1.00	5.00	-0.19	-0.09
**Neuroticism**	1.00	5.00	0.13	-0.51
Anxiety	1.00	5.00	0.10	-0.60
Depression	1.00	5.00	0.20	-0.67
**Openness**	1.40	5.00	-0.34	-0.22
Aesthetics	1.00	5.00	-0.40	-0.64
Ideas	1.20	5.00	-0.37	0.09
**FUD-S**	1.00	6.00	1.56	2.44

Note. N=3,835 users + non-users of Facebook for results regarding the Big Five Inventory (BFI) (sub-)scales; N=2,629 Facebook users for the results regarding the Facebook Use Disorder scale (FUD-S). Scales assessing the broad Big Five factors are bolded.

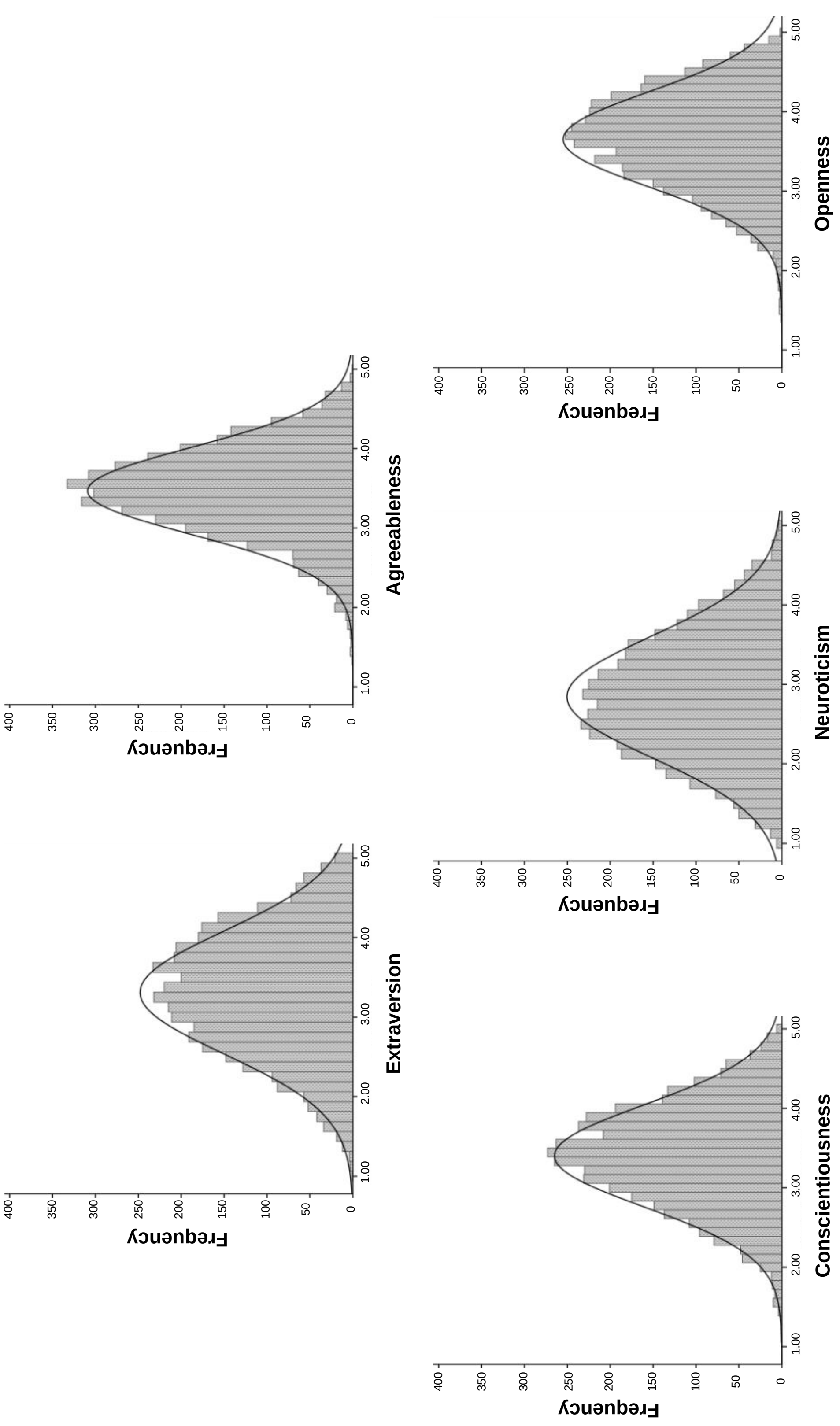

Supplementary Figure 1. Histograms of the distributions of the scales of the BFI. The data of the complete sample of N=3,835 users and non-users of Facebook were used.

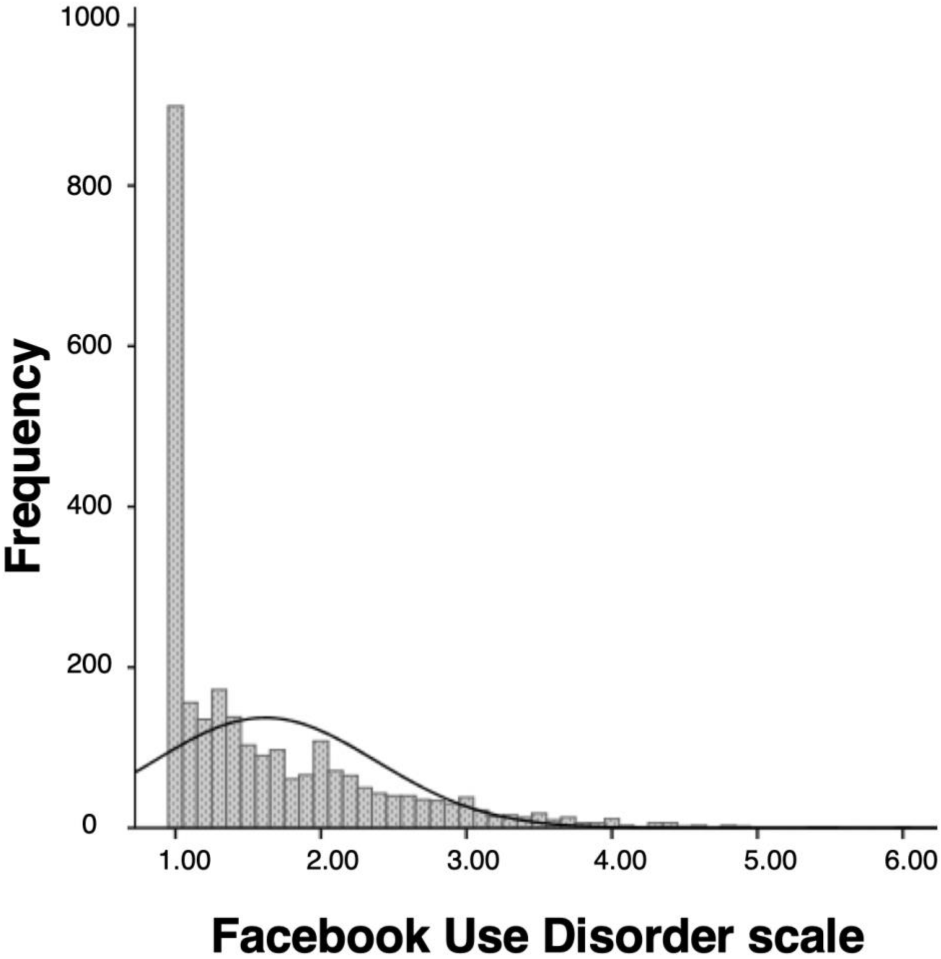

Supplementary Figure 2. Histogram of the distribution of the Facebook Use Disorder scale in the sample of N=2,629 Facebook users.

Reference

Miles J, Shevlin M. *Applying regression and correlation: A guide for students and researchers*. London: Sage; 2001.

Analyses and Results regarding Socio-Demographic Variables

Methods

Associations of age, gender, and education with the variables of interest (BFI (sub-)scales and Facebook use variables) were examined for their potential inclusion as covariates in later analyses.

Therefore, the relationships between age, BFI variables, and the FUD-S were calculated by means of Pearson or Spearman correlations, respectively. Additionally, mean age differences between Facebook users and non-users were tested using Welch's t-test (due to a significant Levene's test).

Independent t-tests were run to examine gender differences in the BFI (for the complete sample). Welch's t-tests were calculated where appropriate. Mann-Whitney U-tests were used to examine gender differences in the FUD-S (Facebook user sample). Chi_2-tests were used to examine the difference in the gender distributions between users and non-users of Facebook.

To test for potential differences in the BFI (sub-)scales between the groups of different educational level, a multivariate ANOVA and ANOVAs for each BFI (sub-)scale separately were conducted in the complete sample. Differences in the FUD-S between groups with different educational level were calculated by means of a Kruskal-Wallis-Test [so far, the analyses on educational background were not registered, but deemed important later on]. Differences in educational level between users and non-users of Facebook were calculated using a Chi_2-test [this analysis was registered].

Results

Age correlated significantly ($p<.05$) with nearly all (sub-)scales of the BFI except the activity subscale of extraversion ($r=.02$, $p=.245$), the altruism ($r=.03$, $p=.078$) and compliance subscales ($r=.01$, $p=.516$) of agreeableness (all other p-values <.003). Age correlated positively with all (sub-)scales except the neuroticism (sub-)scales, which revealed a negative correlation with age. Among the group of Facebook users, age was significantly negatively correlated with the FUD-S ($r_s=-.04$, $p=.049$, $n=2{,}629$). The groups, users versus non-users of Facebook, differed significantly in age ($t(1810.80)=-11.84$, $p<.001$; M(user)=30.50 (SD=10.21) years vs. M(non-users)=35.84 (SD=14.05) years) with non-users being older.

Significant gender differences were found in all (sub-)scales of the BFI (all p-values <.003) except the compliance subscale ($t(3833)=-1.05$, $p=.294$) of agreeableness. Females scored higher than males on all (sub-)scales except the ideas subscale of the factor openness (males > females). Descriptive statistics are presented in Table 1 in the main manuscript. Within the users sample, gender differences in the FUD-S were also found with females scoring higher than males ($U=722471.50$, $p<.001$; M(males)=1.56 (SD=0.75), M(females)=1.70 (SD=0.78)). Additionally, the Chi_2-test revealed that within the Facebook users sample, the male-to-female-ratio was lower (60:40) than in the non-users sample (65:35) ($Chi_2(1)=10.35$, $p=.001$).

Both the MANOVA and the separate ANOVAs carried out to test potential differences in the BFI (sub-)scales between the groups of different educational level revealed several significant results. Perhaps unsurprisingly, no linear trend was observed for any of the BFI (sub-)scales, rendering these relationships difficult to interpret. There was no significant difference in the mean FUD-S score between groups reporting different levels of highest level of education ($Chi_2(6)=3.50$, $p=.744$). As can be seen in Supplementary Table 3, users and non-users differed significantly in terms of highest educational degrees ($Chi_2(6)=47.59$, $p<.001$). This is most likely due to more participants in the users group having a university degree (41.5%) compared to those in the non-users group (36.2%).

Supplementary Table 3

Distribution of highest educational degrees separately for Facebook users and non-users

	users (n=2,629)	non-users (n=1,206)
no graduation	24	35
streamed secondary school for lesser able students	58	46
secondary school leaving certificate	307	177
vocational baccalaureate diploma	162	88
A-level / High school diploma	691	274
university of applied sciences degree	295	149
university degree	1092	437

Note. Educational degrees are translated from the German: no graduation = ohne Schulabschluss, streamed secondary school for lesser able students = Volks-/ Hauptschulabschluss, secondary school leaving certificate = Mittlere Reife, vocational baccalaureate diploma = Fachabitur, A-level / High school diploma = Abitur, university of applied sciences degree = Fachhochschulabschluss, university degree = Hochschulabschluss.

Given the significant results regarding age and gender, both variables were included in the main analyses in the main manuscript to control for possible confounding effects. Despite differences between users and non-users of Facebook in the distributions of highest educational degrees, this variable was not included in the main analyses given the mixed and non-linear findings especially also with respect to associations with the BFI and the FUD-S.
